# Supplementary material for: Evidence for the nuclear import of histones H3.1 and H4 as monomers
Source: EMBO J. 2018 Sep 3;37(19):e98714. doi: 10.15252/embj.201798714 (PMC6166134; doi:10.15252/embj.201798714)
Supplement: Supplementary file 1 — Appendix [file EMBJ-37-e98714-s001.pdf]

Appendix

Table of Contents

Antibodies, plasmids, reagents	2-3
Sequences of constructs used	4-5
Full list of pairwise P-values	6
References	7

Antibodies, plasmids, reagents

Reagent or Resource	Source	Identifier
**Plasmids**		
pEGFP^{TVMVx2}-FRB-OMP25	This study (EGFP vectors derived from pEGFP-C1/C3 series, Clonetech)	N/A
pEGFP-FRB-OMP25	This study	N/A
pH3.1-EGFP^{TVMVx2}-FRB-OMP25	This study	N/A
pH3.1-mCherry^{TVMVx2}-FRB-OMP25	This study	N/A
pH3.1(57-135)-EGFP^{TVMVx2}-FRB-OMP25	This study	N/A
pH3.1(1-56)-EGFP^{TVMVx2}-FRB-OMP25	This study	N/A
pH3.1(31-135)-EGFP^{TVMVx2}-FRB-OMP25	This study	N/A
pH3.1(1-30)-EGFP^{TVMVx2}-FRB-OMP25	This study	N/A
pH3.1(20-135)-EGFP^{TVMVx2}-FRB-OMP25	This study	N/A
pH3.1(1-20)-EGFP^{TVMVx2}-FRB-OMP25	This study	N/A
pH4-EGFP^{TVMVx2}-FRB-OMP25	This study	N/A
pH4(47-102)-EGFP^{TVMVx2}-FRB-OMP25	This study	N/A
pH4(1-46)-EGFP^{TVMVx2}-FRB-OMP25	This study	N/A
pH4(21-102)-EGFP^{TVMVx2}-FRB-OMP25	This study	N/A
pH4(1-20)-EGFP^{TVMVx2}-FRB-OMP25	This study	N/A
pmCherry-FKBP12-TVMV-AI	This study	N/A
pmCherry-FKBP12-TVMV	This study	N/A
pTagBFP-FKBP12-TVMV-AI	This study	N/A
piFP2.0-FKBP12-TVMV-AI	This study	N/A
PCNA-V_HH-TagRFP	Cell Cycle Chromobody® (Chromotek)	Cat: ccr
pEGFP-TEV-sNASP_IRESpuro2	This study	N/A
pEGFP-TEV-ASF1b_IRESpuro2	This study	N/A
pEGFP-TEV-Hat1_IRESpuro2	This study	N/A
pmCherry-NES-ASF1A	This study	N/A
pmCherry-NES-HAT1	This study	N/A
pmCherry-NES-RbAp46	This study	N/A
pmCherry-sNASP-ΔNLS	Subcloned from (Bowman et al., 2016)	N/A
LaminA/C	This study	N/A
pmCherry-sNASP	This study	N/A
pmCherry-C1	Clonetech	Cat: 632524
pmCherry-IPO4	This study	N/A
pmCherry-KPNB1	This study	N/A
pmCherry-IPO11	This study	N/A

MBP-CHAF1B-441-552	This study	N/A
MBP-RbAp46 1-19	This study	N/A
MBP-HAT1-320-420	This study	N/A
ASF1A-GST	(Bowman et al., 2017)	N/A
pETMCN6His-sNASP	(Bowman et al., 2016)	N/A
**Stable cell lines**		
HeLa Kyoto, EGFP-sNASP	This study. Plasmid pEGFP-TEV-sNASP_IRESpuro2 stably integrated. Mycoplasma negative.	N/A
HeLa Kyoto, EGFP-ASF1b	This study. Plasmid pEGFP-TEV-ASF1b_IRESpuro2 stably integrated. Mycoplasma negative.	N/A
HeLa Kyoto, EGFP-HAT1	This study. Plasmid pEGFP-TEV-HAT1_IRESpuro2 stably integrated. Mycoplasma negative.	N/A
**Antibodies**		
Alpha-Tubulin	Sigma	Cat: T9026
ASF1A	Cell Signalling	Cat: C6E10
CHAF1B (CAF1p60)	Sigma	Cat: HPA021679
HAT1	Sigma	Cat: HPA036788
Histone H3	Abcam	Cat: ab1791
Histone H4	Abcam	Cat: ab7311
Lamin A/C	Abcam	Cat: ab108595
NASP	Prof. Andreas Ladurner	N/A
RbAp46	Sigma	Cat: R4154
HRP-goat-anti-mouse	BIO-RAD	Cat: STAR120P
HRP-goat-anti-rabbit	BIO-RAD	Cat: STAR121P
Alexa 488- Goat-anti-mouse	Thermo-Fisher	Cat: A32723
Alexa 568-goat-anti-rabbit	Thermo-Fisher	Cat: A-11036
**Non-general chemicals**		
Fugene HD	Promega	Cat: E2311
Hoechst	New England Biolabs	Cat: 4082S
Paraformaldehyde	Fisher Scientific	Cat: 11490570
Rapamycin	Fisher scientific	Cat: BP2963-1
**Software and Algorithms**		
CellProfiler	doi:10.1186/gb-2006-7-10-r100	http://cellprofiler.org/
ImageJ	doi:10.1038/nmeth.2089	https://imagej.nih.gov/ij/
FIJI	doi:10.1038/nmeth.2019	https://fiji.sc/
RStudio	RStudio, Inc.	https://www.rstudio.com/products/rstudio/download/
Volocity	PerkinElmer	http://cellularimaging.perkinelmer.com/downloads/

Sequences of constructs used

All constructs were verified by Sanger sequencing.

EGFP$^{\mathrm{TVMVx2}}$-FRB-OMP25 (*EGFP*, **TVMVx2**, **FRB**, *OMP25*):

MVSKGEELFTGVVPILVELDGDVNGHKFSVSGEGEGDATYGKLTLKFICTTGKLPVPWPTLVTTLTYGVQCFSRYPDH
MKQHDFFKSAMPEGYVQERTIFFKDDGNYKTRAEVKFEGDTLVNRIELKGIDFKEDGNILGHKLEYNYNSHNVYIMAD
KQKNGIKVNFKIRHNIEDGSVQLADHYQQNTPIGDGPVLLPDNHYLSTQSALSKDPNEKRDHMVLLEFVTAAGITLGM
*DELYKY*SDLEGGSGGSGG**ETVRFQS**GGS**ETVRFQS**GGGGSGGSGGAGGGSGGSGG**VAILWHEMWHEGLEEASR**
LYFGERNVKGMFEVLEPLHAMMERGPQTLKETSFNQAYGRDLMEAQEWCRKYMKSGNVKDLTQAWDLYYHVFR
RIGGTGGSGGTGGSGGSGGSGGTGGS*ELKLRGDGEPSGVPVAVVLLPVFALTLVAVWAFVRYRKQL*

H3.1-EGFP$^{\mathrm{TVMVx2}}$-FRB-OMP25 (H3.1, *EGFP*, **TVMVx2**, **FRB**, *OMP25*):

MARTKQTARKSTGGKAPRKQLATKAARKSAPATGGVKKPHRYRPGTVALREIRRYQKSTELLIRKLPFQRLVREIAQD
FKTDLRFQSSAVMALQEACEAYLVGLFEDTNLCAIHAKRVTIMPKDIQLARRIRGERAGGGSGGGSGGGSGGGALPV
AT*MVSKGEELFTGVVPILVELDGDVNGHKFSVSGEGEGDATYGKLTLKFICTTGKLPVPWPTLVTTLTYGVQCFSRYP*
DHMKQHDFFKSAMPEGYVQERTIFFKDDGNYKTRAEVKFEGDTLVNRIELKGIDFKEDGNILGHKLEYNYNSHNVYIM
ADKQKNGIKVNFKIRHNIEDGSVQLADHYQQNTPIGDGPVLLPDNHYLSTQSALSKDPNEKRDHMVLLEFVTAAGITL
*GMDELYKY*SDLEGGSGGSGG**ETVRFQS**GGS**ETVRFQS**GGGGSGGSGGAGGGSGGSGG**VAILWHEMWHEGLEE**
ASRLYFGERNVKGMFEVLEPLHAMMERGPQTLKETSFNQAYGRDLMEAQEWCRKYMKSGNVKDLTQAWDLYYH
VFRRIGGTGGSGGTGGSGGSGGSGGTGGS*ELKLRGDGEPSGVPVAVVLLPVFALTLVAVWAFVRYRKQL*

H4-EGFP$^{\mathrm{TVMVx2}}$-FRB-OMP25 (H4, *EGFP*, **TVMVx2**, **FRB**, *OMP25*):

MSGRGKGGKGLGKGGAKRHRKVLRDNIQGITKPAIRRLARRGGVKRISGLIYEETRGVLKVFLENVIRDAVTYTEHAK
RKTVTAMDVVYALKRQGRTLYGFGGGGGSGGGSGGGSGGGVAT*MVSKGEELFTGVVPILVELDGDVNGHKFSVSG*
EGEGDATYGKLTLKFICTTGKLPVPWPTLVTTLTYGVQCFSRYPDHMKQHDFFKSAMPEGYVQERTIFFKDDGNYKT
RAEVKFEGDTLVNRIELKGIDFKEDGNILGHKLEYNYNSHNVYIMADKQKNGIKVNFKIRHNIEDGSVQLADHYQQNTP
*IGDGPVLLPDNHYLSTQSALSKDPNEKRDHMVLLEFVTAAGITLGMDELYKY*SDLEGGSGGSGG**ETVRFQS**GGS**ETV**
RFQSGGGGSGGSGGAGGGSGGSGG**VAILWHEMWHEGLEEASRLYFGERNVKGMFEVLEPLHAMMERGPQTLK**
ETSFNQAYGRDLMEAQEWCRKYMKSGNVKDLTQAWDLYYHVFRRIGGTGGSGGTGGSGGSGGSGGTGGS*ELKL*
RGDGEPSGVPVAVVLLPVFALTLVAVWAFVRYRKQL

EGFP-FRB-OMP25 (*EGFP*, **FRB**, *OMP25*):

MVSKGEELFTGVVPILVELDGDVNGHKFSVSGEGEGDATYGKLTLKFICTTGKLPVPWPTLVTTLTYGVQCFSRYPDH
MKQHDFFKSAMPEGYVQERTIFFKDDGNYKTRAEVKFEGDTLVNRIELKGIDFKEDGNILGHKLEYNYNSHNVYIMAD
KQKNGIKVNFKIRHNIEDGSVQLADHYQQNTPIGDGPVLLPDNHYLSTQSALSKDPNEKRDHMVLLEFVTAAGITLGM
*DELYKY*SDLEGGSGGSGG**VAILWHEMWHEGLEEASRLYFGERNVKGMFEVLEPLHAMMERGPQTLKETSFNQAY**
GRDLMEAQEWCRKYMKSGNVKDLTQAWDLYYHVFRRIGGTGGSGGTGGSGGSGGSGGTGGS*ELKLRGDGEPS*
GVPVAVVLLPVFALTLVAVWAFVRYRKQL

mCherry-FKBP12-TVMV-AI (*mCherry*, **FKBP12**, *TVMV*, **AI**):

MVSKGEEDNMAIIKEFMRFKVHMEGSVNGHEFEIEGEGEGRPYEGTQTAKLKVTKGGPLPFAWDILSPQFMYGSKA
YVKHPADIPDYLKLSFPEGFKWERVMNFEDGGVVTVTQDSSLQDGEFIYKVKLRGTNFPSDGPVMQKKTMGWEASS
ERMYPEDGALKGEIKQRLKLKDGGHYDAEVKTTYKAKKPVQLPGAYNVNIKLDITSHNEDYTIVEQYERAEGRHSTGG
*MDELYK*SGLRSRAQGGSGGSGG**VQVETISPGDGRTFPKRGQTCVVHYTGMLEDGKKFDSSRDRNKPFKFMLGKQ**
EVIRGWEEGVAQMSVGQRAKLTISPDYAYGATGHPGIIPPHATLVFDVELLKLEGGSGGSGGSGGSGGSGG*SKALL*
KGVRDFNPISACVCLLENSSDGHSERLFGIGFGPYIIANQHLFRRNNGELTIKTMHGEFKVKNSTQLQMKPVEGRDIIVI
KMAKDFPPFPQKLKFRQPTIKDRVCMVSTNFQQKSVSSLVSESSHIVHKEDTSFWQHWITTKDGQCGSPLVSIIDGNI
*LGIHSLTHTTNGSNYFVEFPEKFVATYLDAADGWCKNWKFNADKISWGSFILWEDAPEDFM*SGLVPRGVGR**EYVRF**
APGS

mCherry-FKBP12-TVMV (*mCherry*, **FKBP12**, *TVMV*):

MVSKGEEDNMAIIKEFMRFKVHMEGSVNGHEFEIEGEGEGRPYEGTQTAKLKVTKGGPLPFAWDILSPQFMYGSKA
YVKHPADIPDYLKLSFPEGFKWERVMNFEDGGVVTVTQDSSLQDGEFIYKVKLRGTNFPSDGPVMQKKTMGWEASS
ERMYPEDGALKGEIKQRLKLKDGGHYDAEVKTTYKAKKPVQLPGAYNVNIKLDITSHNEDYTIVEQYERAEGRHSTGG
*MDELYK*SGLRSRAQGGSGGSGG**VQVETISPGDGRTFPKRGQTCVVHYTGMLEDGKKFDSSRDRNKPFKFMLGKQ**
EVIRGWEEGVAQMSVGQRAKLTISPDYAYGATGHPGIIPPHATLVFDVELLKLEGGSGGSGGSGGSGGSGG*SKALL*
KGVRDFNPISACVCLLENSSDGHSERLFGIGFGPYIIANQHLFRRNNGELTIKTMHGEFKVKNSTQLQMKPVEGRDIIVI
KMAKDFPPFPQKLKFRQPTIKDRVCMVSTNFQQKSVSSLVSESSHIVHKEDTSFWQHWITTKDGQCGSPLVSIIDGNI
*LGIHSLTHTTNGSNYFVEFPEKFVATYLDAADGWCKNWKFNADKISWGSFILWEDAPEDFM*SG

TagBFP-FKBP12-TVMV-AI (*TagBFP*, **FKBP12**, *TVMV*, **AI**):

MSELIKENMHMKLYMEGTVDNHHFKCTSEGEGKPYEGTQTMRIKVVEGGPLPFAFDILATSFLYGSKTFINHTQGIPD
FFKQSFPEGFTWERVTTYEDGGVLTATQDTSLQDGCLIYNVKIRGVNFTSNGPVMQKKTLGWEAFTETLYPADGGLE
GRNDMALKLVGGSHLIANIKTTYRSKKPAKNLKMPGVYYVDYRLERIKEANNETYVEQHEVAVARYCDLPSKLGHKLN
SGLRSRAQGGSGGSGG**VQVETISPGDGRTFPKRGQTCVVHYTGMLEDGKKFDSSRDRNKPFKFMLGKQEVIRGW**
EEGVAQMSVGQRAKLTISPDYAYGATGHPGIIPPHATLVFDVELLKLEGGSGGSGGSGGSGGSGG*SKALLKGVRD*

FNPISACVCLLENSSDGHSERLFGIGFGPYIIANQHLFRRNNGELTIKTMHGEFKVKNSTQLQMKPVEGRDIIVIKMAKD
FPPFPQKLKFRQPTIKDRVCMVSTNFQQKSVSSLVSESSHIVHKEDTSFWQHWITTKDGQCGSPLVSIIDGNILGIHSL
*THTTNGSNYFVEFPEKFVATYLDAADGWCKNWKFNADKISWGSFILWEDAPEDFM*SGLVPRGVGR**EYVRFAP**GS

IFP2.0-FKBP12-TVMV-AI (*TagBFP*, **FKBP12**, *TVMV*, **AI**):

MARDPQPFFPPLYLGGPEITTENCEREPIHIPGSIQPHGALLTADGHSGEVLQVSLNAATFLGQEPTVLRGQTLAALLP
DQWPALQTALPPGCQDALQYRATLDWPAAGHLSLTVHRVAELLILEFEPTEAWDSIGPHALRNAMFALESAPNLRAL
AEVATQTVRELSGFDRVMLYKFAPDATGEVIAEARREGMQAYLGHRFPASTTPAQARALYTRHLLRLTADTRAAAVP
LDPVLNPQTNAPTPLGGAVLRATSPMHMQYLRNMGVGSSLSVSVVVGGQLWGLIVCHHQTPYVLPPDLRTTLEYLG
*RLLSL*QVQRKEASGLRSRAQGGSGGSGG**VQVETISPGDGRTFPKRGQTCVVHYTGMLEDGKKFDSSRDRNKPFKF**
MLGKQEVIRGWEEGVAQMSVGQRAKLTISPDYAYGATGHPGIIPPHATLVFDVELLKLEGGSGGSGGSGGSGGSG
GSKALLKGVRDFNPISACVCLLENSSDGHSERLFGIGFGPYIIANQHLFRRNNGELTIKTMHGEFKVKNSTQLQMKPV
EGRDIIVIKMAKDFPPFPQKLKFRQPTIKDRVCMVSTNFQQKSVSSLVSESSHIVHKEDTSFWQHWITTKDGQCGSPL
VSIIDGNILGIHSLTHTTNGSNYFVEFPEKFVATYLDAADGWCKNWKFNADKISWGSFILWEDAPEDFMSGLVPRGVG
R**EYVRFAP**GS

Sequence information for EGFP chaperone fusions

EGFP-sNASP (*EGFP*, **TEV**, **sNASP**):

MVSKGEELFTGVVPILVELDGDVNGHKFSVSGEGEGDATYGKLTLKFICTTGKLPVPWPTLVTTLTYGVQCFSRYPDH
MKQHDFFKSAMPEGYVQERTIFFKDDGNYKTRAEVKFEGDTLVNRIELKGIDFKEDGNILGHKLEYNYNSHNVYIMAD
KQKNGIKVNFKIRHNIEDGSVQLADHYQQNTPIGDGPVLLPDNHYLSTQSALSKDPNEKRDHMVLLEFVTAAGITLGM
*DELYKS*GLRSRAQASNSAVDGTGSG**ENLYFQG**H**MAMESTATAAVAAELVSADKIEDVPAPSTSADKVESLDVDSE**
AKKLLGLGQKHLVMGDIPAAVNAFQEAASLLGKKYGETANECGEAFFFYGKSLLELARMENGVLGNALEGVHVEE
EEGEKTEDESLVENNDNIDETEGSEEDDKENDKTEEMPNDSVLENKSLQENEEEEIGNLELAWDMLDLAKIIFKRQE
TKEAQLYAAQAHLKLGEVSVESENYVQAVEEFQSCLNLQEQYLEAHDRLLAETHYQLGLAYGYNSQYDEAVAQFS
KSIEVIENRMAVLNEQVKEAEGSSAEYKKEIEELKELLPEIREKIEDAKESQRSGNVAELALKATLVESSTSGFTPGG
GGSSVSMIASRKPTDGASSSNCVTDISHLVRKKRKPEEESPRKDDAKKAKQEPEVNGGSGDAVPSGNEVSENMEE
EAENQAESRAAVEGTVEAGATVESTAC

EGFP-ASF1b (EGFP, **TEV**, **ASF1b**):

MVSKGEELFTGVVPILVELDGDVNGHKFSVSGEGEGDATYGKLTLKFICTTGKLPVPWPTLVTTLTYGVQCFSRYPDH
MKQHDFFKSAMPEGYVQERTIFFKDDGNYKTRAEVKFEGDTLVNRIELKGIDFKEDGNILGHKLEYNYNSHNVYIMAD
KQKNGIKVNFKIRHNIEDGSVQLADHYQQNTPIGDGPVLLPDNHYLSTQSALSKDPNEKRDHMVLLEFVTAAGITLGM
*DELYKS*GLRSRAQASNSAVDGTGSG**ENLYFQG**H**MAKVSVLNVAVLENPSPFHSPFRFEISFECSEALADDLEWKIIY**
VGSAESEEFDQILDSVLVGPVPAGRHMFVFQADAPNPSLIPETDAVGVTVVLITCTYHGQEFIRVGYYVNNEYLNPEL
RENPPMKPDFSQLQRNILASNPRVTRFHINWDNNMDRLEAIETQDPSLGCGLPLNCTPIKGLGLPGCIPGLLPENSM
DCI

EGFP-HAT1 (*EGFP*, **TEV**, **HAT1**):

MVSKGEELFTGVVPILVELDGDVNGHKFSVSGEGEGDATYGKLTLKFICTTGKLPVPWPTLVTTLTYGVQCFSRYPDH
MKQHDFFKSAMPEGYVQERTIFFKDDGNYKTRAEVKFEGDTLVNRIELKGIDFKEDGNGHILGHKLEYNYNSHNVYIM
ADKQKNGIKVNFKIRHNIEDGSVQLADHYQQNTPIGDGPVLLPDNHYLSTQSALSKDPNEKRDHMVLLEFVTAAGITL
*GMDELYKS*GLRSRAQASNSAVDGTGSG**ENLYFQG**H**MAGFGAMEKFLVEYKSAVEKKLAEYKCNTNTAIELKLVRF**
PEDLENDIRTFFPEYTHQLFGDDETAFGYKGLKILLYYIAGSLSTMFRVEYASKVDENFDCVEADDVEGKIRQIIPPGF
CTNTNDFLSLLEKEVDFKPFGTLLHTYSVLSPTGGENFTFQIYKADMTCRGFREYHERLQTFLMWFIETASFIDVDDE
RWHYFLVFEKYNKDGATLFATVGYMTVYNYYVYPDKTRPRVSQMLILTPFQGQGHGAQLLETVHRYYTEFPTVLDI
TAEDPSKSYVKLQDFVLVKLCQDLPCFSREKLMQGFNEDMAIEAQQKFKINKQHARRVYEILRLLVTDMSDAEQYR
SYRLDIKRRLISPYKKKQRDLAKMRKCLRPEELTNQMNQIEISMQHEQLEESFQELVEDYRRVIERLAQE

<u>Full list of pairwise P-values</u>

The full list of P-values for Figure 3D are as follows.

Group A	Group B	P-value
H3.1, 30 min	H3.1, 1 hour	0.724
H3.1, 30 min	H3.1, 2.5.hours	$< 1x10^{-07}$
H3.1, 30 min	H3.1, 5.hours	$< 1x10^{-07}$
H4, 30 min	H4, 1.hr	0.222
H4, 30 min	H4, 2.5.hr	$< 1x10^{-07}$
H4, 30 min	H4, 5.hr	$< 1x10^{-07}$

Pairwise P-values

The full list of P-values for Figure 4C is as follows:

Antibody probe	Group A	Group B	P-value
H3	EGFP	H3	$6.51\ x10^{-10}$
	EGFP	H4	0.0421
H4	EGFP	H3	$1.25\ x10^{-04}$
	EGFP	H4	$8.07\ x10^{-15}$
ASF1A	EGFP	H3	0.336
	EGFP	H4	$7.33\ x10^{-03}$
HAT1	EGFP	H3	0.181
	EGFP	H4	0.57
RbAp46	EGFP	H3	0.0138
	EGFP	H4	0.318
NASP	EGFP	H3	$5.71\ x10^{-03}$
	EGFP	H4	1.0

The full list of P-values for Figure 4F is as follows:

mCherry probe	Group A	Group B	P-value
ASF1A-NES	EGFP	H3	$6.54\ x10^{-10}$
	EGFP	H4	$1.38\ x10^{-09}$
HAT1-NES	EGFP	H3	$1.30\ x10^{-05}$
	EGFP	H4	$6.49\ x10^{-10}$
RbAp46-NES	EGFP	H3	0.102
	EGFP	H4	$6.54\ x10^{-10}$
sNASP-ΔNLS	EGFP	H3	$8.07\ x10^{-15}$
	EGFP	H4	0.949

Figure 5B

Histone tether	Group A	Group B	P-value
H3	mCherry	IPO4	$4.03\ x10^{-15}$
	mCherry	IPO11	$1.33\ x10^{-03}$
	mCherry	KPNB1	$9.98\ x10^{-04}$
H4	mCherry	IPO4	$6.54\ x10^{-10}$
	mCherry	IPO11	$3.98\ x10^{-04}$
	mCherry	KPNB1	$5.61\ x10^{-13}$

Appendix References

Bowman, A., Koide, A., Goodman, J.S., Colling, M.E., Zinne, D., Koide, S., and Ladurner, A.G. (2017). sNASP and ASF1A function through both competitive and compatible modes of histone binding. Nucleic Acids Res *45*, 643-656.

Bowman, A., Lercher, L., Singh, H.R., Zinne, D., Timinszky, G., Carlomagno, T., and Ladurner, A.G. (2016). The histone chaperone sNASP binds a conserved peptide motif within the globular core of histone H3 through its TPR repeats. Nucleic Acids Res *44*, 3105-3117.
