# Supplementary material for: Evidence for the nuclear import of histones H3.1 and H4 as monomers
Source: EMBO J. 2018 Sep 3;37(19):e98714. doi: 10.15252/embj.201798714 (PMC6166134; doi:10.15252/embj.201798714)
Supplement: Supplementary file 2 — Expanded View Figures PDF [file EMBJ-37-e98714-s002.pdf]

Expanded View Figures

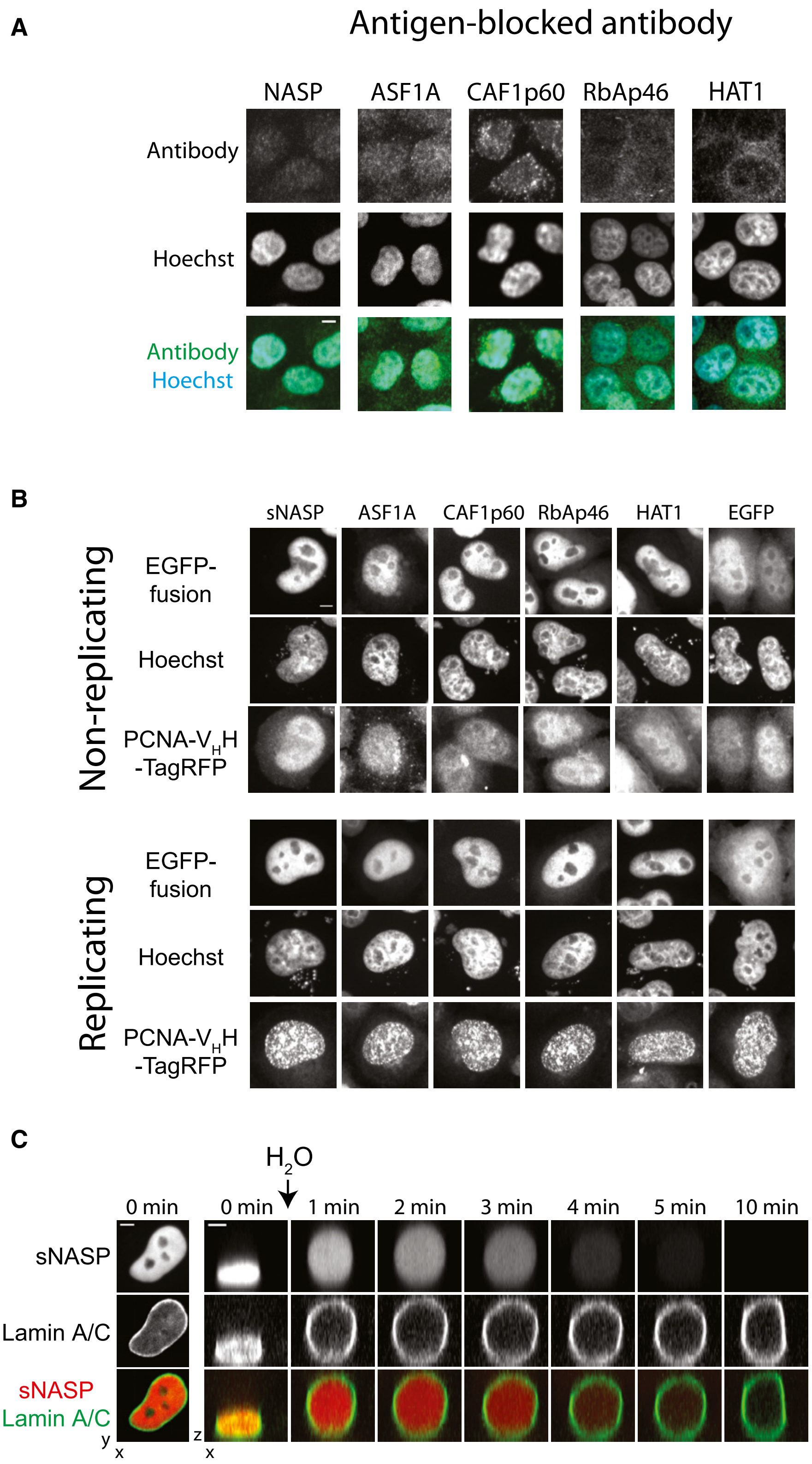

Figure EV1. Related to Fig 1.

A Immunofluorescence using antibodies pre-blocked with their immunogen. In each case, the specific nuclear fluorescence was decreased to background levels. Scale bar represents 5 μm.

B Cellular location of transiently expressed EGFP-histone chaperone fusions in replicating and non-replicating cells. Scale bars represent 5 μm.

C Time series of a cell expressing mCherry-sNASP and EGFP-Lamin A/C undergoing hypotonic lysis. Scale bar represents 5 μm.

© 2018 The Authors

Figure EV2. Related to Fig 2.

A Cleavage site deletion mutant. The auto-inhibited TVMV protease is recruited to the tethered EGFP, but is unable to release it due to the absence of a TVMV cleavage site separating EGFP from its tether. Scale bar represents 5 μm.

B Auto-inhibitory (AI) deletion mutant. Deletion of the AI domain from the mCherry-FKBP12-TVMV-AI construct results in constitutively active protease that cleaves the tethered EGFP independent of recruitment. Scale bar represents 5 μm.

C Histogram of a time course for EGFP or H4-EGFP release. Pixels from the whole cell (grey), the cytosol (blue) and the nucleus (red) plotted for the cells are shown in Fig 2E. Whilst the modal value from the whole cell significantly shifts to the right after the release of EGFP, with overlapping cytosol and nuclear signals, the modal value for H4-EGFP remains at a similar level, whilst the nuclear and cytosolic signals form discrete populations. Grey lines represent modal pixel values before rapamycin addition. Scale bar represents 5 μm.

 © 2018 The Authors

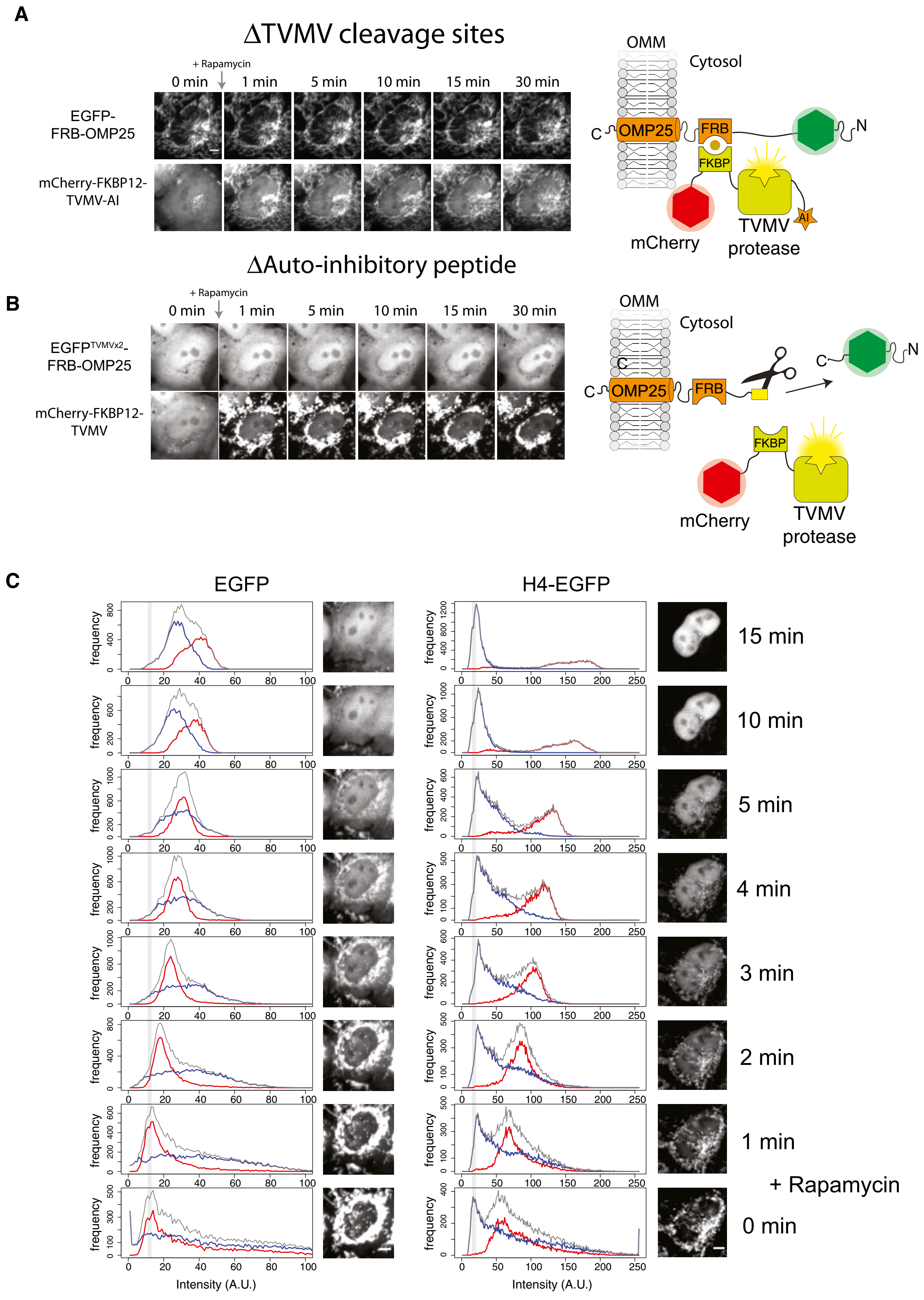
A
ΔTVMV cleavage sites
+ Rapamycin
0 min
1 min
5 min
10 min
15 min
30 min
EGFP-FRB-OMP25
mCherry-FKBP12-TVMV-AI
OMM
Cytosol
OMP25
FRB
FKBP
mCherry
TVMV protease
AI
ΔAuto-inhibitory peptide
B
EGFP^TVMVx2-FRB-OMP25
mCherry-FKBP12-TVMV
C
EGFP
H4-EGFP
frequency
Intensity (A.U.)
15 min
10 min
5 min
4 min
3 min
2 min
1 min
+ Rapamycin
0 min

Figure EV2.

Figure EV3. Related to Fig 4.

A An example cell expressing tethered EGFP and probed with an anti-H3 antibody, demonstrating the image processing workflow. Raw images are shown on top, and background-corrected images shown below. The manually partitioned cytosolic region encompassing the mitochondrial network is outlined in red. A bisection of the cell is indicated in the images and the corresponding profiles of each channel shown in the third column. The Pearson's correlation coefficient of the raw and corrected images is shown, demonstrating the artifactually high colocalisation from the gradient of background staining in the raw image coinciding with the preference of mitochondria to reside around the microtubule organising centre of the cell, juxtaposed to the nucleus.

B Representative images for cells quantified are shown in Fig 4C. Scale bar represents 5 μm.

C Representative images from cells quantified are shown in Fig 4E. Scale bar represents 5 μm.

 © 2018 The Authors

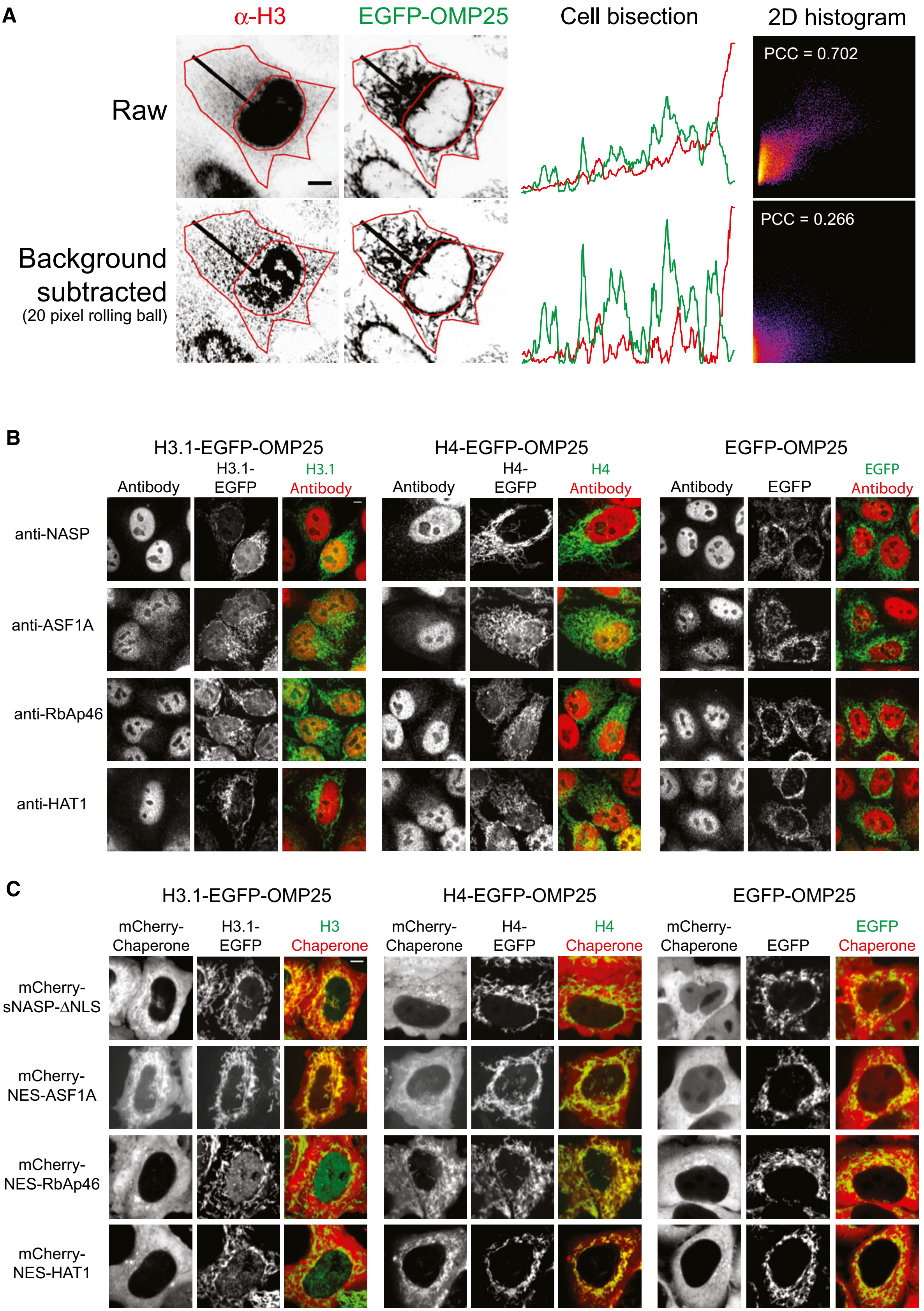
A
α-H3
EGFP-OMP25
Cell bisection
2D histogram
Raw
PCC = 0.702
Background subtracted
(20 pixel rolling ball)
PCC = 0.266
B
H3.1-EGFP-OMP25
H4-EGFP-OMP25
EGFP-OMP25
Antibody
H3.1-EGFP
H3.1 Antibody
H4-EGFP
H4 Antibody
EGFP
EGFP Antibody
anti-NASP
anti-ASF1A
anti-RbAp46
anti-HAT1
C
mCherry-Chaperone
H3 Chaperone
H4 Chaperone
EGFP Chaperone
mCherry-sNASP-ΔNLS
mCherry-NES-ASF1A
mCherry-NES-RbAp46
mCherry-NES-HAT1

Figure EV3.

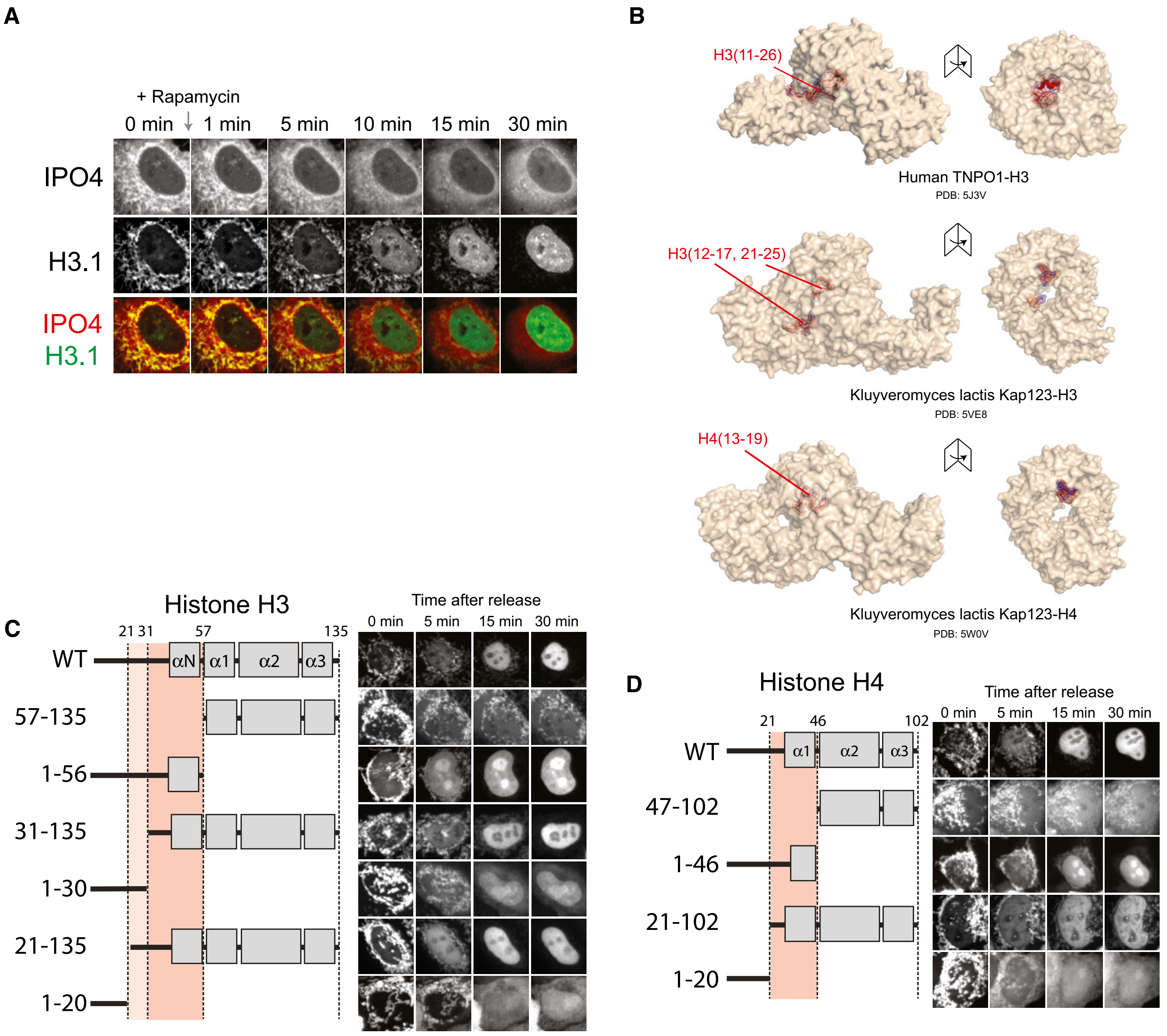

Figure EV4. Related to Fig 5.

A A second representative cell showing a time course of H3.1-EGFP release from its mitochondrial tether after co-transfection with mCherry-IPO4.
B Crystal structures of importin-β proteins bound to H3 and H4 peptides.
C Analysis of H3.1 truncations for nuclear import. The region important for import is shaded in red.
D Analysis of H4 truncations for nuclear import. The regions important for import are shaded in red.

© 2018 The Authors

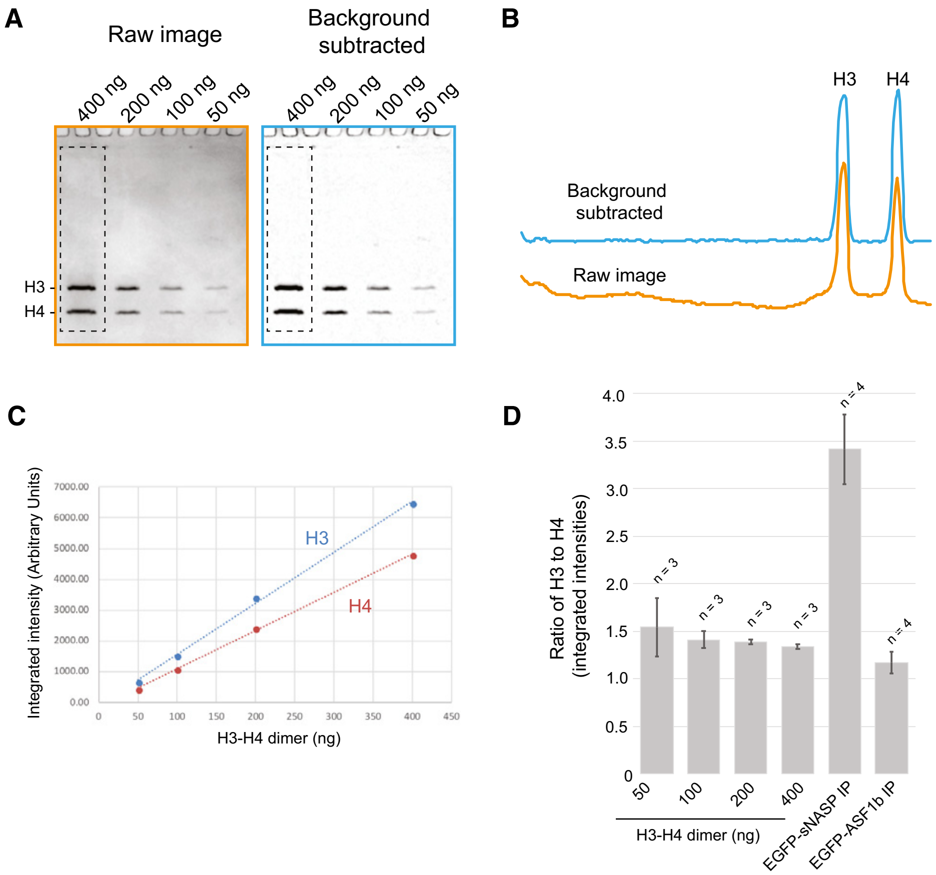

Figure EV5. Related to Fig 6.

A Serial dilutions for H3-H4 quantification. 50–400 ng of H3-H4 dimer was separated by SDS–PAGE and stained with Coomassie. Raw images were subject to background subtraction using an 18 pixel rolling ball algorithm in ImageJ.

B Profiles of the hatched lanes shown in (A).

C Plot of the H3-H4 dimer concentration versus staining intensity from the gel shown in (A). A linear relationship is shown between 50 and 400 ng titration points.

D Plot of the intensity ratios for H3 and H4 from three independent experiments. Error bars indicate the standard deviation. Ratios from eGFP-sNASP and eGFP-ASF1b immunoprecipitations from the main text are shown alongside for comparison. Note, these values are not normalised, whereas in Fig 6C, the values are normalised to ASF1b.

© 2018 The Authors
