# Supplementary material for: Evidence for the nuclear import of histones H3.1 and H4 as monomers
Source: EMBO J. 2018 Sep 3;37(19):e98714. doi: 10.15252/embj.201798714 (PMC6166134; doi:10.15252/embj.201798714)
Supplement: Supplementary file 4 — Movie EV2 [file EMBJ-37-e98714-s004.zip › Legend_Movie_EV2.docx]

**Movie Legends**

**Movie EV2. Related to Figure 2E.** RAPID-release of H3.1-EGFP. Time point ‘0 min’ is pre-rapamycin addition.
