# Supplementary material for: Evidence for the nuclear import of histones H3.1 and H4 as monomers
Source: EMBO J. 2018 Sep 3;37(19):e98714. doi: 10.15252/embj.201798714 (PMC6166134; doi:10.15252/embj.201798714)
Supplement: Supplementary file 8 — Source Data for Figure 1 [file EMBJ-37-e98714-s007.pdf]

Source Data Figure 1

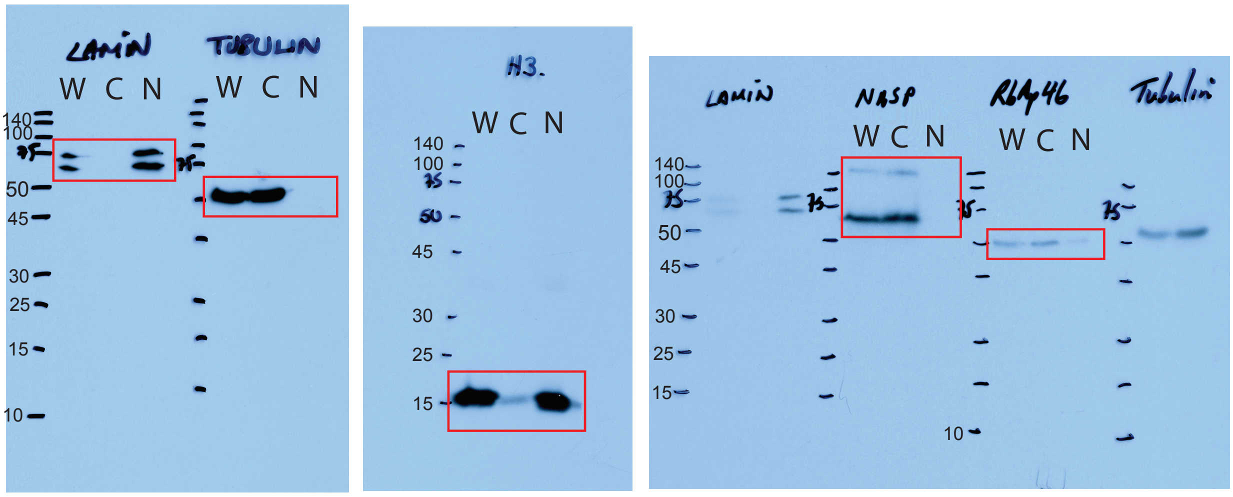

1 minute exposure

10 minute exposure

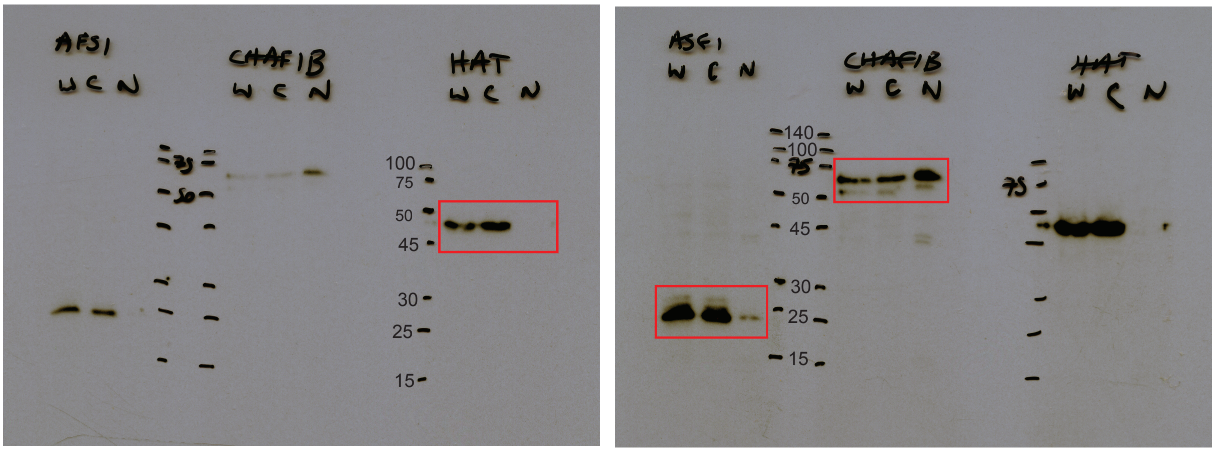

Relating to Figure 1A. Full immunoblots. W - whole cell extract, C - cytosolic extract, N - nuclear extract. Numbers indicate molecular weight markers. Red boxes indicate the regions used to create the composite figure.
