# Supplementary material for: Evidence for the nuclear import of histones H3.1 and H4 as monomers
Source: EMBO J. 2018 Sep 3;37(19):e98714. doi: 10.15252/embj.201798714 (PMC6166134; doi:10.15252/embj.201798714)
Supplement: Supplementary file 9 — Source Data for Figure 6 [file EMBJ-37-e98714-s008.pdf]

Source Data Figure 6

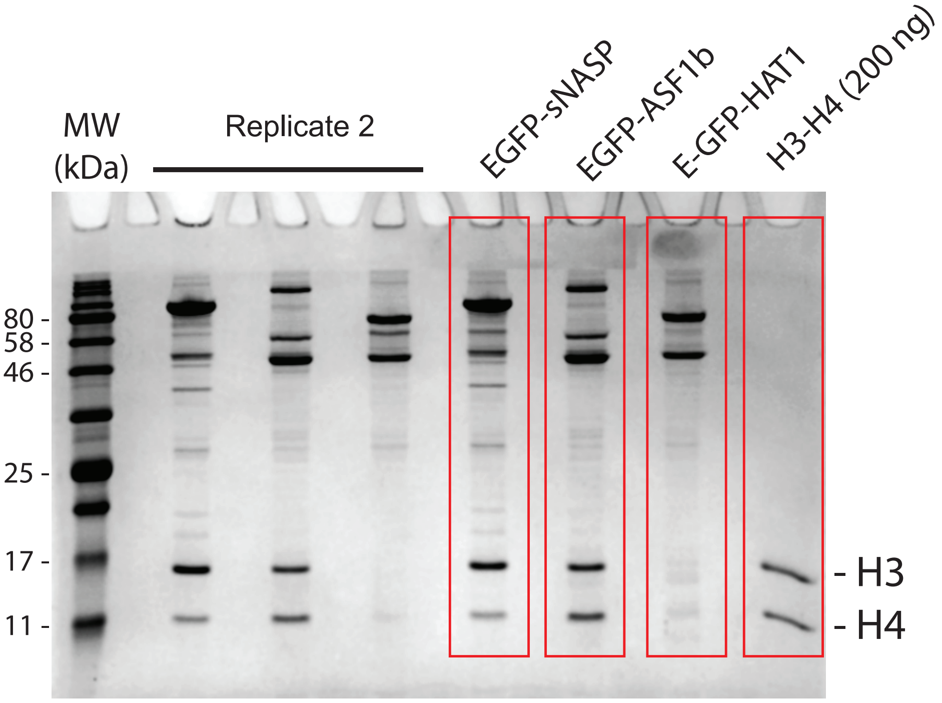

Related to Figure 6B. Full coomassie stained, SDS-PAGE gel used to create Figure 6B. Regions used to create the composite image are boxed in red.
